# Advantages of Broad-Spectrum Influenza mRNA Vaccines and Their Impact on Pulmonary Influenza

**DOI:** 10.3390/vaccines12121382

**Published:** 2024-12-07

**Authors:** Ziqi Cheng, Junfeng Ma, Chenyan Zhao

**Affiliations:** 1 National Engineering Laboratory for AIDS Vaccine, School of Life Sciences, Jilin University, Changchun 130012, China; chengzq21@mails.jlu.edu.cn; 2 Division of HIV/AIDS and Sex-Transmitted Virus Vaccines, National Institutes for Food and Drug Control (NIFDC), Beijing 102629, China

**Keywords:** broad-spectrum influenza vaccine, mRNA Vaccine mRNA, pulmonary influenza

## Abstract

Influenza poses a significant global health challenge due to its rapid mutation and antigenic variability, which often leads to seasonal epidemics and frequent outbreaks. Traditional vaccines struggle to offer comprehensive protection because of mismatches with circulating viral strains. The development of a broad-spectrum vaccine is therefore crucial. This paper explores the potential of mRNA vaccine technology to address these challenges by providing a swift, adaptable, and broad protective response against evolving influenza strains. We detail the mechanisms of antigenic variation in influenza viruses and discuss the rapid design and production, enhanced immunogenicity, encoding of multiple antigens, and safety and stability of mRNA vaccines compared to traditional methods. By leveraging these advantages, mRNA vaccines represent a revolutionary approach in influenza prevention, potentially offering broad-spectrum protection and significantly improving global influenza management and response strategies.

## 1. Introduction

Influenza, an acute respiratory infection, annually represents a major global health challenge due to its severe respiratory symptoms [1,2]. Annually, influenza results in approximately 3 to 5 million cases of severe illness worldwide and 290,000 to 650,000 respiratory deaths, according to the World Health Organization [3,4]. Severe outcomes are more common among high-risk groups, including the elderly, young children, and individuals with chronic health conditions such as asthma, diabetes, or heart disease. These populations are more susceptible to severe respiratory complications like pneumonia and acute respiratory distress syndrome (ARDS) [5,6].

Clinically, influenza presents with a spectrum of symptoms ranging from mild respiratory distress to severe pneumonia and ARDS, which can lead to fatal outcomes if not promptly and effectively managed. The burden of influenza is not only limited to its direct health impact but also extends to significant economic costs related to hospitalizations and reduced productivity during epidemic seasons [7,8].

The disease is highly transmissible, and is predominantly spread through respiratory droplets. Influenza viruses are categorized into four types—A, B, C, and D—based on the antigenic properties of their nucleoproteins (NPs) and matrix (M) protein. Type A viruses, which can infect both humans and various animals, are particularly problematic due to their ability to undergo rapid genetic reassortment, leading to antigenic drift and increased cross-species transmission risks. This rapid evolution complicates the continual adaptation of vaccine formulations, a challenge that traditional vaccines frequently struggle to address effectively [9]. The virus’s inherent rapid mutation rate, coupled with the surge in infections during the winter and spring, complicates timely vaccine updates. As a result, influenza not only places a significant burden on public health systems but also has a substantial impact on the global economy.

Without targeted treatments for influenza, vaccination is the primary preventive strategy. Traditional vaccine types include inactivated viruses and live attenuated viruses, which primarily work by inducing neutralizing antibodies and stimulating cellular immunity. However, these conventional vaccine strategies often struggle to keep pace with the rapid antigenic drift and shift of influenza viruses, resulting in significant discrepancies between vaccine strains and circulating strains [10,11]. Such mismatches compromise vaccine effectiveness and underscore the urgent need for more adaptable and responsive vaccine technologies.

Recent advancements in mRNA vaccine technology represent a significant milestone in the fight against viral infections [12]. These vaccines have shown high efficacy and rapid deployment, notably in response to influenza. mRNA vaccines provide a dynamic, scalable, and adaptable platform that addresses the limitations of traditional influenza vaccines [13,14]. Their capability to swiftly generate specific antigens in response to evolving pathogens, combined with their potential to induce robust and broad immune responses, makes them particularly effective against rapidly mutating viruses like influenza. By directly encoding viral antigens, mRNA vaccines can be quickly updated to reflect changes in viral strains, ensuring broader protection against diverse influenza variants and enhancing global management and response strategies for influenza [15,16,17].

This review examines the diversity and mechanisms of variation in influenza viruses, emphasizing the unique challenges they present. It outlines mRNA vaccine technology, highlighting its transformative potential for influenza prevention. We focus on the advantages of mRNA vaccines, particularly their broad-spectrum efficacy against pulmonary influenza, which enhances resilience to both seasonal and pandemic influenza threats, ultimately improving public health outcomes.

## 2. Influenza Virus Diversity and Mechanisms of Variation

### 2.1. Classification of Influenza Viruses

**Figure 1:**
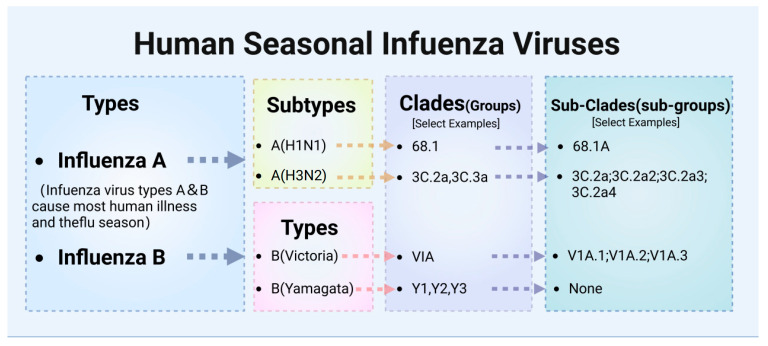
Classification of Human Influenza Viruses. This diagram illustrates the hierarchical classification of human seasonal influenza viruses, showing the division into types, subtypes, lineages, clades, and sub-clades. Influenza A is categorized into subtypes A(H1N1) and A(H3N2), while Influenza B is divided into Victoria and Yamagata lineages. The diagram further details examples of clades and sub-clades for each subtype, providing a clear framework for understanding the genetic diversity within seasonal influenza viruses.

Influenza viruses belong to the Orthomyxoviridae family and are characterized as enveloped, segmented, negative-sense RNA viruses. They are classified into four types: A, B, C, and D. These types are differentiated by antigenic variations in their M proteins and NPs [18] (Figure 1). Types A and B (Influenza A Viruses, IAVs, and Influenza B Viruses, IBVs) are most relevant to human health, with Influenza A being particularly virulent and responsible for all historic influenza pandemics and most seasonal epidemics [19,20].

Influenza A is characterized by its extensive genetic diversity, with 18 recognized hemagglutinin (HA) subtypes (H1-H18) and 11 neuraminidase (NA) subtypes (N1-N11), making A(H1N1) and A(H3N2) the most prevalent [21]. The A(H1N1) subtype, known for its role in several pandemics, including the 2009 swine flu pandemic, features clades such as 6B.1, subdividing into sub-clades like 6B.1A. This genetic structure enables detailed tracking of virus evolution and aids in the adaptation of vaccines. The A(H3N2) subtype, often linked to severe seasonal flu cases, includes clades such as 3C.2a and 3C.3a, with sub-clades extending to 3C.2a1 through 3C.2a4, demonstrating the dynamic mutation process and challenges it presents for vaccine formulation [19]. In 2021, the H5N1 avian influenza virus, specifically clade 2.3.4.4b, underwent a significant geographic expansion, impacting wild birds and domestic poultry across Asia, Europe, and Africa, and eventually reaching North America by year’s end. This spread contributed to one of the largest recorded outbreaks of H5N1, affecting 37 countries during the 2021–2022 epidemic wave [22]. These viruses are characterized by their considerable genetic diversity and a high frequency of reassortment, traits that became particularly pronounced as they spread across continents. The continuous evolution, broadening host range, and expanding geographic reach of the clade 2.3.4.4b viruses significantly elevate their potential to trigger a pandemic [23].

Influenza B viruses primarily infect humans and generally cause outbreaks that are less extensive than those caused by Type A viruses [24]. However, this pattern is not without exceptions. Over the past two decades, several seasons have seen Type B viruses cause significant morbidity, at times comparable to Type A [20,25,26]. For instance, the B(Victoria) lineage, known for its significant genetic diversity within clades such as V1A (including sub-clades V1A.1, V1A.2, and V1A.3), was responsible for severe outbreaks during the 2001–2002 season that notably affected children and the widespread European outbreak in 2015 [27]. Furthermore, during the 2019–2020 season, the B(Victoria) lineage accounted for about 50% of all identified influenza viruses, marking a significant departure from the typical dominance of Type A. This trend was also observed in Australia in 2015, where 62% of circulating viruses were Type B [26]. The B(Yamagata) lineage, including clades Y1, Y2, and Y3, has shown no confirmed circulation since 2020 and is thus considered extinct, leading to its exclusion from vaccine formulations starting in the 2024–2025 flu season, to better align with the current epidemiological landscape and enhance vaccine effectiveness [28]. Additionally, influenza B and A viruses demonstrate distinct antigenic and genetic behaviors. Influenza B evolves more conservatively, exhibiting slower antigenic changes [29]. This slower rate of antigenic change means that even significant genetic mutations may not promptly affect its antigenicity. This decoupling often results in discrepancies between antigenic cartography with genetic analysis results [30]. These inconsistencies can complicate the direct correlation needed for effective vaccine strain selection, emphasizing the necessity for specialized strategies to accurately track and respond to influenza B [31].

Influenza C viruses typically cause milder and less frequent illnesses in humans [13]. Unlike A and B, Influenza C does not exhibit a range of subtypes but instead has a single serotype, primarily due to the limited variability of its hemagglutinin-esterase-fusion (HEF) protein. This protein combines the functions of HA and NA found in Influenza A and B, contributing to a more stable antigenic profile, leading to fewer antigenic changes [20,25]. The simpler genomic structure of Influenza C, with only seven RNA segments and no separate NA segment, further limits its antigenic diversity and evolutionary potential [25,32]. Consequently, the limited antigenic variation of Influenza C typically only results in sporadic cases and minor outbreaks, not widespread epidemics. However, ongoing surveillance and research are crucial to detect any significant changes in the antigenic properties of Influenza C. Such changes could potentially alter the virus’s disease profile and public health impact.

Influenza D viruses lack recognized subtypes and possess a unique hemagglutinin-like protein that significantly differs from those found in other influenza viruses. This unique protein results in limited antigenic similarity and cross-reactivity with these human-infecting viruses. Consequently, immune responses developed against Influenza A, B, or C are unlikely to recognize or effectively combat Influenza D. However, they are significant in veterinary medicine due to their role in respiratory diseases among cattle, which could have economic impacts on agriculture [20]. These viruses have been associated with respiratory diseases in cattle, sparking ongoing research focused on their potential for cross-species transmission and the broader implications for animal health [33]. Ongoing research is focused on the potential for Influenza D to adapt and on cross-species transmission, with implications for animal health and biosecurity. This includes monitoring its antigenic shifts and drifts to understand how it might evolve to more effectively infect new hosts, including potentially humans.

The diversity of influenza viruses is largely attributed to their ability to infect a wide array of host animals, encompassing various bird and mammal species [34]. This extensive host range enables genetic recombination and antigenic shifts, key mechanisms posing continual challenges to developing consistently effective influenza vaccines.

### 2.2. Structure of Influenza A Viruses

**Figure 2:**
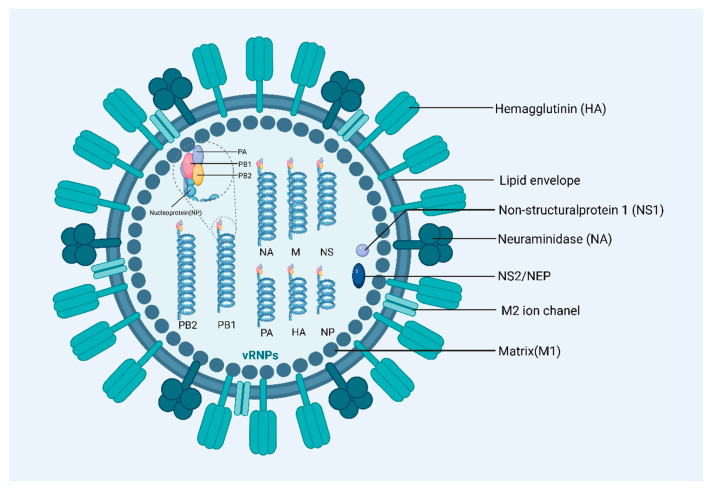
Structrue of Influenza A Viruses. Influenza A virus structure, highlighting its lipid envelope with embedded HA and NA glycoproteins for cell attachment and release. Inside, the matrix (M1) protein supports the envelope, with M2 ion channels regulating internal pH. The core contains eight segments of negative-strand RNA, forming ribonucleoprotein complexes with nucleoproteins, essential for viral replication. HA, haemagglutinin; M1, matrix protein; M2, membrane protein; NA, neuraminidase; NS1, nonstructural protein 1; PA, polymerase acidic protein; PB1, polymerase basic protein 1; PB2, polymerase basic protein 2.

Influenza A viruses are characterized by their pleomorphic nature, displaying both spherical and filamentous forms. These particles typically range from 80 to 120 nm in diameter, with filamentous variants extending beyond 300 nm. This variability in shape and size complicates structural analysis and impacts how these viruses are studied and understood in virology [35]. The genome of Influenza A viruses consists of eight segments of negative-strand RNA, encoding up to seventeen proteins, crucial for various functions within the virus lifecycle. Central among these are the surface proteins: HA, NA, and matrix-2 protein (M2) [20,36]. HA is the most abundant surface protein of the influenza virus and plays a crucial role in mediating viral entry into host cells. Approximately 60% of anti-influenza antibodies recognize HA, making it a key target for vaccine development [37]. NA aids in the release of newly formed virus particles from host cells by cleaving sialic acid residues, which facilitates the spread of the infection [38]. M2 functions as a transmembrane proton channel that modulates the internal pH of the virion, essential for viral uncoating during cell entry (Figure 2).

The virus’s genome also comprises eight RNA segments that encode the RNA polymerase complex (PB1, PB2, and PA) necessary for viral replication. The viral replication is driven by an RNA-dependent RNA polymerase contained within the viral ribonucleoprotein (RNP) complex, utilizing a positive-sense complementary RNA intermediate. This process is supported by the RNA polymerase complex proteins—B1, PB2, and PA—which are integral to the replication and transcription of the viral genome. Additionally, Influenza A viruses produce non-structural proteins, such as NS1 and NS2/NEP, which play key roles in modulating host cellular functions to optimize conditions for viral replication and assembly [39]. NS1 is particularly involved in evading host immune responses, while NS2/NEP assists in the export of viral ribonucleoproteins from the nucleus to the cytoplasm, facilitating subsequent steps in the viral life cycle.

### 2.3. Mechanisms of Antigenic Variation

Influenza’s rapid antigenic evolution presents a substantial challenge in vaccine design by complicating the prediction of dominant strains each flu season [40]. This evolution, driven by mechanisms like antigenic drift and shift, enables the virus to effectively evade host immune responses, contributing to its persistent impact on global health [41].

#### 2.3.1. Antigenic Drift

Antigenic drift, identified by Maurice Hilleman in the 1940s, is a primary evolutionary mechanism for influenza viruses [40]. Antigenic drift occurs in both influenza A and influenza B viruses. It involves gradual genetic mutations primarily affecting HA and NA surface proteins—HA facilitates viral entry, and NA aids in viral release [42]. These mutations are facilitated by an error-prone RNA-dependent RNA polymerase that lacks proofreading capabilities [40]. Consequently, the virus can evade pre-existing immunity by altering antigenic sites recognized by the host immune system [43,44]. The mutation process itself does not result in new dominant strains; rather, new strains of the virus randomly emerge and may possess a fitness advantage in environments where the population has existing immunity. Over time, such variants can become dominant, as they are less recognizable to immune systems primed by older strains [42,45].

The persistence and pace of antigenic drift are influenced by the duration of the epidemic and the intensity of the immune response [46]. Longer influenza seasons provide more opportunities for the virus to replicate and mutate, while strong immune responses in the population increase the selection pressure for the virus to evolve novel antigens to evade immunity [47,48]. This interplay dictates the selection pressure for novel antigens, leading to continuous antigenic evolution that poses significant challenges for vaccine design.

#### 2.3.2. Antigenic Shift

Antigenic shift, also discovered by Hilleman, is a significant genetic transformation mechanism in influenza viruses that occurs through the process of reassortment [41]. This event transpires when two different influenza A virus strains co-infect a single cell, often in a human or an intermediate host like pigs or chickens. This process enables these viruses to exchange gene segments, particularly those coding for the HA and NA proteins [49,50]. Such genetic mixing can produce a novel influenza virus with potentially unique surface antigens. When these antigens differ significantly from parental strains, the new virus may evade immunity established in populations by previous strains. For instance, during late 2023 and early 2024, a novel reassortant strain emerged in poultry within Cambodia and Vietnam, combining surface proteins from clade 2.3.2.1c with internal genes from the more recent clade 2.3.4.4b, demonstrating substantial genetic exchange [22]. Similarly, during the 2022/2023 influenza season, a significant reassortment among circulating H3N2 viruses was noted, with about 88% of sequenced genomes showing signs of genetic mixing with other clades [51]. However, it is crucial to recognize that not all new viruses display entirely novel antigens. For instance, the HA antigen of the 2009 H1N1 pandemic influenza virus (Ca09) showed substantial antigenic similarity to its parental strains. Studies revealed that the HA gene of the novel H1N1 virus shared 93.2% to 93.4% nucleotide homology with swine H1N1 viruses isolated from 2006 to 2007 in the United States [52]. This similarity played a critical role in the population’s existing immunity and the overall impact of the pandemic [53]. Such findings underscore that while reassortment can introduce significant genetic changes, the antigenic properties of the resultant virus might not always be drastically different from those of its progenitors, thus complicating public health predictions [54]. Additionally, intragenic reassortment and recombination within gene segments can further complicate the evolutionary pathway of these viruses, leading to new variants with altered pathogenicity or immune evasion capabilities. For example, potential recombination events between the PB2 and HA genes of H3N2 viruses suggest intricate mechanisms of viral evolution that contribute to the continual challenge of predicting and controlling influenza outbreaks [55].

#### 2.3.3. Other Factors

Antigenic variation in influenza viruses is influenced not only by antigenic drift and shift but also by a range of ecological and genomic factors. Interactions between various host species, including humans, pigs, and birds, play a pivotal role in the evolution of influenza viruses [56]. While migratory birds are instrumental in dispersing influenza A subtypes across regions, their migration routes typically do not cover intercontinental distances [57]. Conversely, human activities, particularly the international trade of live birds and poultry products, play a significant role in the global spread of avian influenza. For example, the introduction of H5N1 and other H5NX strains into the United States has primarily been attributed to human-mediated transfers rather than natural bird migration [58]. This situation illustrates the intricate interaction between natural avian behaviors and human activities in determining the epidemiology of influenza [34,59].

The zoonotic potential of influenza arises from its ability to jump from animals to humans, often with little warning, posing risks of new pandemics. This is particularly true for avian influenza strains, which typically infect the gastrointestinal (G.I.) tract of birds and are transmitted through fecal-oral routes [60]. Such transmission enables the virus to be shed in feces, elevating the risk of zoonotic transmission in environments where humans are in close contact with infected animals, such as live poultry markets or farms [61]. Additionally, avian influenza’s capacity to infect the G.I. tract allows it to spread via water and surfaces, enhancing its environmental stability and potential for wider dissemination. The resilience of these viruses to the acidic conditions and digestive enzymes in the G.I. tract further aids their survival outside the host, increasing the likelihood of interspecies transmission [60]. Meanwhile, pigs serve as mixing vessels for new strains, providing a critical environment where reassortment of influenza viruses from different hosts can occur, potentially leading to the emergence of novel variants. Additionally, the immune system of each host species exerts selective pressure on influenza viruses, driving mutations [62,63]. Adaptations to a new host can result in changes to viral antigens that confer advantages in the new host environment but may alter the virus’s antigenic properties relative to the original host [64].

The zoonotic potential of influenza means that viruses circulating in animal populations can unpredictably jump to humans, potentially triggering new pandemics with scant forewarning [65]. For example, influenza viruses, particularly avian strains, commonly infect the gastrointestinal (G.I.) tract. This mode of transmission among birds through fecal-oral routes allows the virus to be shed in feces significantly raises the risk of zoonotic transmission, especially in settings like live poultry markets or farms where humans frequently contact infected animals or contaminated environments. What’s more, avian influenza can infect the gastrointestinal (G.I.) tract, enabling it to spread through water and on surfaces, thereby increasing its environmental stability and potential for broader dissemination. The ability of these viruses to withstand the acidic conditions and digestive enzymes within the G.I. tract enhances their survival outside the host.

The genomic structure of influenza viruses also significantly shapes their pathogenic potential and public health impact. Types A and B possess eight RNA segments, while types C and D have seven, each capable of encoding multiple viral proteins [66]. This segmented RNA structure adds to the virus’s complexity [67]. Mutations in one part of the viral genome can influence the effects of mutations elsewhere, a phenomenon known as epistasis. These interactions can impact the virus’s fitness, antigenicity, and virulence [68] For example, modifications in the HA protein may compensate for fitness costs imposed by mutations in other proteins, thus enhancing the virus’s ability to spread and maintain pathogenicity [68,69].

Simultaneously, the antigenic thrift theory provides a nuanced view of how influenza viruses evade population immunity. According to this theory, population immunity targets epitopes of limited variability (ELVs) [40,70]. These ELVs do not undergo significant changes, making them stable targets for the immune system. However, demographic shifts—such as changes in birth and death rates—alter the landscape of immunity within a population. As individuals with immunity to historical strains pass away and new susceptible individuals are born, previously suppressed viruses can re-emerge once collective immunity to their ELVs diminishes. This dynamic complicates vaccine design and public health strategies, as it requires not only keeping pace with evolving strains but also accounting for the cyclic re-emergence of older strains due to changes in population immunity.

### 2.4. Limitations of Classical Influenza Vaccines

Several types of influenza vaccines have been in use for many years, including whole virus, split-virion, subunit, live attenuated virus, and virosome vaccines. Each of these vaccines employs different strategies to elicit an immune response and each has a well-established history of use in preventing influenza (Table 1). Influenza vaccines are crucial but face challenges due to the virus’s rapid evolution and broad host range, including humans, birds, and pigs [71]. These hosts can harbor and propagate diverse viral strains, often leading to mismatches between vaccine formulations and circulating strains. Such mismatches, a result of the lengthy lead time required for strain selection and vaccine manufacturing, can significantly reduce vaccine effectiveness. Additionally, newer approaches, such as synthetic long-peptide vaccines, which are designed to enhance T-cell responses against more conserved regions of the virus, are being explored to overcome these challenges, although they are not yet widely in use [72].

Since the 1940s, inactivated influenza vaccines have been a mainstay in flu prevention. Traditionally, these vaccines are produced using egg-based methods, where live viruses are inoculated into fertilized chicken eggs and allowed to replicate. Once ample replication has occurred, the virus is harvested, inactivated by chemical or physical methods, and purified to create the vaccine. This egg-based method has been the standard due to its efficacy in producing adequate virus quantities for vaccine development [73,74]. However, for strains that grow poorly in eggs or to accommodate individuals with egg allergies, cell culture-based production methods are also utilized [75,76,77]. Both methods stimulate a robust immune response by presenting viral antigens without the risk of infection.

Live attenuated influenza vaccines (LAIVs), which target key influenza strains such as A(H1N1), A(H3N2), and B (Victoria), are usually produced using egg-based techniques as well. These vaccines are low in virulence but can still replicate transiently within the body post-vaccination, offering a realistic immune challenge without causing illness [78,79]. However, there is a minor risk associated with LAIVs that they might revert to a more virulent form due to genetic mutations or reassortment with wild-type viruses [80,81].

Aside from attenuated vaccines, virosomes are a type of vaccine delivery system that utilizes virus-like particles. Virosomes effectively deliver vaccine antigens to the immune system by mimicking the natural infection process of viruses, which enhances the immune response without causing disease [82,83,84]. However, due to the complex manufacturing process, the cost may be higher than more traditional vaccines, limiting their widespread use.

DNA vaccines represent another approach, introducing genes encoding influenza proteins into the body to induce an immune response. While historically challenging in eliciting strong immunity in humans, recent advancements in delivery technologies and adjuvants have begun to improve their effectiveness significantly.

Recombinant vaccinia virus vaccines, which utilize the insertion of foreign genes into the vaccinia virus genome, have demonstrated potential in providing broad-spectrum immunity against various influenza strains [85,86]. The application of vaccinia virus vectors in human vaccines, however, is subject to intense scientific examination. A significant concern is the potential impact of pre-existing immunity to the vaccinia virus, which may be acquired through previous smallpox vaccinations or from other recombinant vaccines. This existing immunity could potentially diminish the effectiveness of new vaccinia-based vaccines. Additionally, the development of polyvalent vaccines, designed to protect against multiple strains of influenza, introduces intricate scientific and technical challenges. These vaccines need to effectively integrate antigens from various strains into a single vaccine formulation, which must remain stable and elicit a strong immune response without compromising safety.

To mitigate the issues arising from antigenic variability and the need for rapid vaccine updates, ongoing research aims to reduce the production times and increase the adaptability of vaccines. This extended timeline allows for potential mismatches between the vaccine and circulating strains if the virus mutates or if unexpected strains emerge after strain selection [63]. Such mismatches may necessitate supplementary measures like antiviral drugs [87]. Enhancing the alignment between vaccines and circulating viral strains is crucial, especially given the potential for sudden emergence of new strains that can lead to public health crises. Effective public health strategies must also consider the logistics of vaccine distribution, public perceptions of vaccine safety, and the challenges of co-circulating pathogens, which can complicate diagnosis and treatment.

**Table 1:** Advantages and disadvantages of various vaccines.

	Vaccine Type	Synthesis	Advantages	Disadvantages	Reference
Inactivated Vaccines	Synthetic Long-Peptide Vaccine Inactivated	Peptides synthetically produced to match segments of viral proteins.	Specific immune response targeting, can be designed to enhance T-cell response, lower risk of autoimmunity.	Complex manufacturing process, may require adjuvants to enhance effectiveness, limited long-term data.	[88]
	Whole-Virus Vaccine	Virus grown in culture, then killed using heat or chemicals to inactivate it.	Stable, well-understood, can provoke a strong immune response, long track record of safety.	Risk of incomplete virus inactivation, cold chain storage required, possible allergic reactions.	[77,89,90]
	Split-Virion Vaccine	Virus grown in culture, then physically broken up to remove genetic material, retaining only antigenic proteins.	Reduced risk of infection compared to whole-virus vaccines, better safety profile with high immunogenicity.	May require adjuvants to enhance immunogenicity, not as robust immune response as live vaccines.	[75,79,91,92]
	Subunit Vaccine	Only specific viral proteins or protein fragments used, excluding other viral components.	Highly specific immune response, less risk of side effects from viral proteins, can be used in immunocompromised individuals.	May require multiple doses and adjuvants, potential for lower immunogenicity compared to whole-virus vaccines.	[76,92,93,94]
Live Attenuated Vaccines	Live Attenuated Virus Vaccine	Virus grown in culture and genetically modified to lose virulence but keep immunogenic properties.	Strong and long-lasting immune response, often requires fewer boosters, mimics natural infection.	Risk of causing disease in immunocompromised individuals, potential for reversion to virulence, cold chain storage required.	[78,79,80,81,89,95]
Viral Vector Vaccines	Virosome Vaccine	Viral antigens incorporated into lipid vesicles, mimicking virus structure without genetic material.	Targeted delivery system that can enhance immune response, reduced risk of infection compared to live vaccines.	Complex manufacturing process, cost may be higher than more traditional vaccines, limited data on long-term efficacy.	[82,83,84]
Nucleic Acid Vaccines	mRNA Vaccine	Produced synthetically using a DNA template to make mRNA that encodes a viral protein.	Rapid development, high efficacy, adjustable formulation, induces both humoral and cellular immunity.	Cold chain storage requirements, shorter track record, potential for immune reaction.	[96,97,98]
	DNA Vaccine	Plasmids containing genes of interest synthesized; these are used to transfect cells and induce an immune response.	Can induce a broad range of immune responses, stable and relatively easy to manufacture, not temperature sensitive.	Concerns about integration into host DNA, variable immune response effectiveness, still under development.	[88,99]
	circRNA Vaccine	Circular RNA molecules synthesized to encode for antigens, leveraging the stability and expression efficiency of circRNA.	Can induce potent immune responses, versatile platform with potential for fewer side effects, stability advantages over mRNA.	Newer technology with limited data, manufacturing challenges, regulatory hurdles as a novel technology.	[100,101]
Recombinant Virus Vaccines	Recombinant Vaccinia Virus Vaccine	Vaccinia virus engineered to express influenza virus proteins (e.g., NP, M2e).	Induces both humoral and cellular immune responses, can be engineered for multiple antigens, effective for cross-strain protection.	Risk of reversion to pathogenic forms in some cases, pre-existing immunity may reduce efficacy in certain populations, cold chain storage may be required.	[85,86]

## 3. Overview of mRNA Vaccine Technology

### 3.1. mRNA Vaccine Principles and Mechanisms

**Figure 3:**
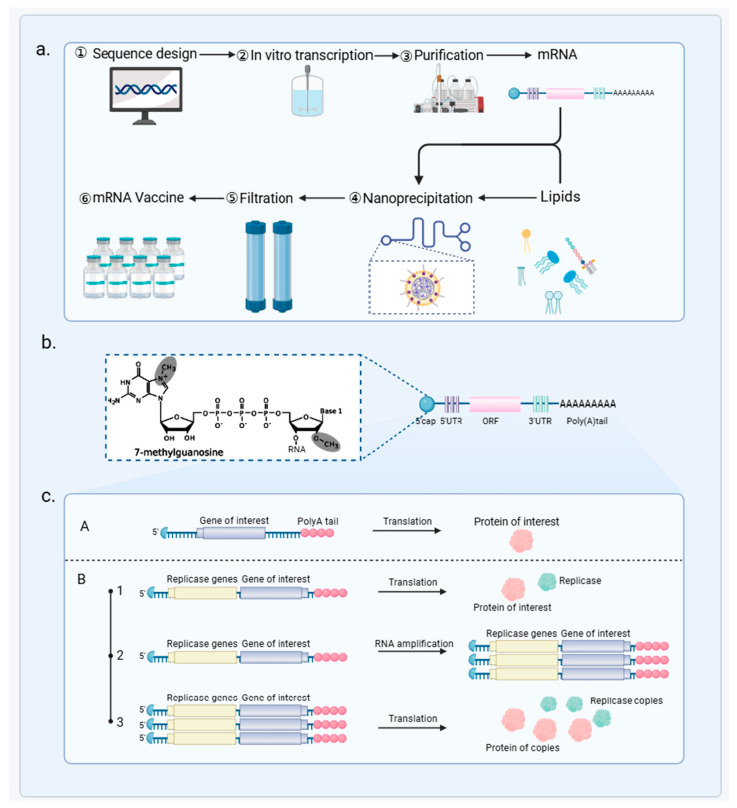
Overview of mRNA Vaccine Development and Function. (**a**) The production of mRNA vaccines starts with sequence design, followed by in vitro transcription, purification, and nanoprecipitation with lipids to form lipid nanoparticles. The final product undergoes filtration to ensure vaccine quality. (**b**) The structure of the mRNA includes a 7-methylguanosine cap, 5′ and 3′ untranslated regions (UTRs), an open reading frame (ORF) coding for the protein of interest, and a stabilizing Poly(A) tail. (**c**) Two mechanisms of protein production are shown: (A) direct translation of the mRNA into the target protein, and (B) replicase-mediated amplification, where replicase genes first produce replicase proteins, leading to RNA amplification and enhanced protein production.

mRNA vaccine technology provides a rapid response to influenza by quickly adapting to viral mutations (Figure 3). The production process initiates with the generation of mRNA from a DNA template using in vitro transcription. Then, the mRNA undergoes capping and polyadenylation, processes that are essential for mRNA stability and translational efficacy [102]. This enhanced mRNA is encapsulated in lipid nanoparticles (LNPs), which protect it from degradation and facilitate efficient cellular uptake [103,104]. Each batch of these vaccines is subjected to stringent quality control checks to ensure its safety, purity, and potency.

The mRNA vaccines against influenza come in two main types: self-amplifying mRNA (saRNA) and non-replicating mRNA [105]. Non-replicating mRNA vaccines are composed of fully processed mRNA, which includes a cap, a poly(A) tail, and untranslated regions (UTRs) [106,107]. They are synthesized from a linear DNA template using bacteriophage RNA polymerases such as T7, T3, or Sp6. This mature mRNA structure mimics that of eukaryotic cellular mRNA, which is pivotal for vaccine stability and function [108,109].

Self-amplifying mRNA (saRNA) vaccines are an advanced type of vaccine technology that includes a segment of the alphavirus genome within the mRNA structure [110,111]. This unique inclusion capitalizes on the alphavirus’s natural ability to produce multiple copies of itself inside the host cell. By integrating this property, saRNA vaccines enhance the replication of the mRNA once inside the host’s cells [112]. This not only increases the amount of antigen produced but also prolongs the duration of antigen expression. By amplifying the amount of antigen produced, saRNA vaccines effectively activate various components of the immune system, including dendritic cells, T cells, and B cells [113]. This comprehensive immune activation is especially beneficial in combating rapidly mutating pathogens like influenza, as it can generate a robust response that adapts to viral changes.

Research is also exploring circular RNAs (circRNAs) for their potential in vaccine development. Due to their covalently closed loop structure, circRNAs exhibit enhanced stability compared to linear RNAs, which might allow for lower vaccine dosages to be effective [114]. These vaccines can encode antigens and act as adjuvants to strengthen immune responses, crucial for T cell activation [115]. However, circRNA vaccines are still in early development stages.

### 3.2. Advantages of mRNA Vaccines as Broad-Spectrum Influenza Vaccines

Although they require specific storage conditions, the scalability and anticipated reduction in production costs as the technology advances further highlight their potential as a cost-effective solution in the field of vaccinology [97,116] (Table 2).

#### 3.2.1. Rapid Vaccine Design and Production

mRNA vaccines are highly effective against influenza due to their rapid adaptability and efficient production process [126,127]. Traditional vaccines often face delays due to the complexities of egg-based production and adaptation challenges [128]. Meanwhile, mRNA vaccines can be quickly updated to match evolving influenza strains. This flexibility depends mainly on factors like RNA length, nucleotides, capping chemistry, and purification methods [129,130]. Additionally, the production process is largely independent of the RNA sequence, which allows for rapid customization of vaccines to effectively and swiftly address new influenza strains. This capability far exceeds that of synthetic long-peptide and DNA vaccines [131,132,133]. Once the sequence of a target antigen is identified, production can start immediately, highlighting the potential of mRNA vaccines not just for managing seasonal flu but also for pandemic preparedness. Their rapid response, cost-effectiveness, and scalability make mRNA vaccines a crucial tool in combating both existing and emerging infectious diseases.

#### 3.2.2. Encoding Multiple Antigens

By incorporating sequences that encode multiple viral antigens, mRNA vaccines can maintain their efficacy across a wide array of virus variants. This feature allows them to be rapidly adapted to target multiple antigens from different influenza strains, effectively responding to the virus’s quick evolutionary changes. For example, a vaccine encoding 20 different HA antigens has shown extensive protection across diverse influenza strains, suggesting the potential for universal vaccines [134]. Researchers like Arevalo et al. have developed multivalent mRNA vaccines that offer broad protection by including antigens from multiple strains [135]. However, the formulation of such vaccines encounters practical and immunological challenges. Administered in small volumes, typically less than 1 mL, vaccines must balance antigen quantity without overwhelming the available dose volume. Overloading a vaccine with multiple antigens can lead to reduced immunogenicity of each component due to volume constraints and potential antigen competition, where the immune response to one or some antigens can be diminished—a phenomenon known as negative interference. This occurs when the presence of multiple antigens in a vaccine leads to reduced antibody responses to one or more of those antigens, complicating the effectiveness of multivalent formulations [136,137]. To mitigate these effects, antigen selection and prioritization are critical. By carefully choosing which antigens to include, manufacturers can optimize the immune response to ensure effective protection against the targeted strains. Despite these challenges, the flexibility and rapid manufacturability of mRNA vaccines remain major advantages [129].

Additionally, advancements in mRNA technology now allow for targeting both HA and NA epitopes, improving immune responses and reducing viral evasion. Studies by Mazunina et al. and Xiong et al. demonstrate that mRNA vaccines can target virus-conserved regions or use chimeric HA constructs to elicit broadly neutralizing antibodies against several influenza subtypes [138,139]. A prominent example of this technology in action is Moderna’s mRNA-1010, a quadrivalent vaccine that protects against two influenza A viruses (A/H1N1 and A/H3N2) and two influenza B viruses (B/Yamagata and B/Victoria), adhering to WHO recommendations. This illustrates how mRNA vaccines can effectively cover a broad spectrum of influenza viruses by integrating multiple antigens [129,135]. Additionally, single doses of RNActive vaccines encoding IAV HA, NP, and NA proteins have produced protective immunity in mice, ferrets, and pigs when administered intradermally [140]. Similar results were seen with intravenous administration of PR8 H1N1 IAV HA-encoding mRNA in lipid complexes, which elevated T-cell activation in mice [141].

Additionally, studies have shown that mRNA vaccines can elicit cross-reactive immune responses, enhancing their protection against multiple influenza strains, as evidenced by a pentavalent mRNA vaccine that effectively guards against various influenza B virus strains [142].

#### 3.2.3. Enhanced Immunogenicity

**Figure 4:**
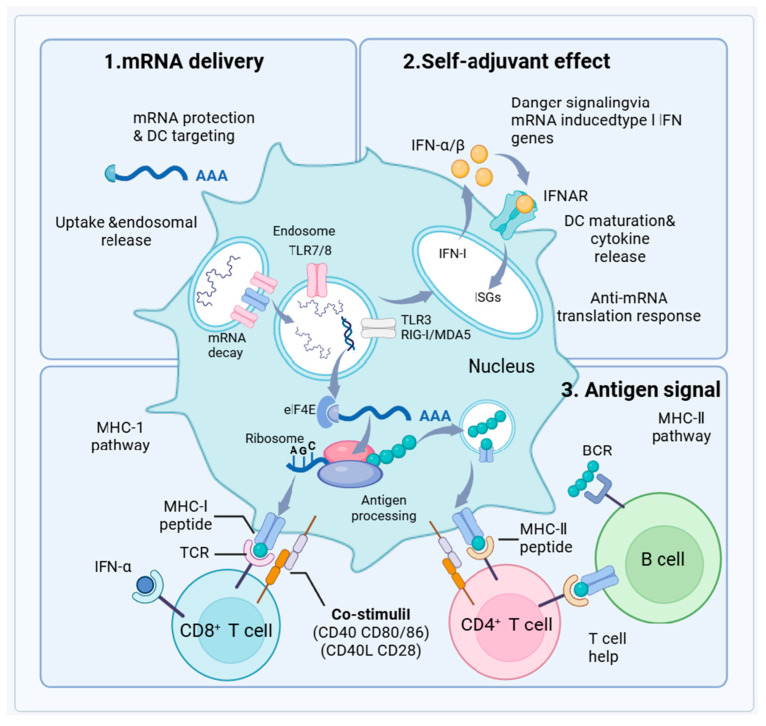
Mechanisms of mRNA Vaccine Efficacy. This diagram depicts the integral processes of mRNA vaccine effectiveness including mRNA delivery with dendritic cell targeting and endosomal uptake, self-adjuvant effects via type I interferon gene induction enhancing dendritic cell responses, and antigen signaling involving MHC molecule presentation and T cell activation. These mechanisms collectively foster both an immediate immune defense and the development of lasting immunity through memory cells.

mRNA vaccines, encapsulated within LNPs, are designed for enhanced stability and effective cellular uptake [143]. This encapsulation protects the mRNA from enzymatic degradation until it reaches the interior of host cells. Once injected, the mRNA is taken up by dendritic cells (DCs), specialized antigen-presenting cells [144]. Inside these cells, the mRNA exits the endosomes and enters the cytoplasm where it is translated into viral antigens by the host’s ribosomes (Figure 4).

The process mimics natural infection by producing viral proteins directly inside the host cells, which are then displayed on the cell surface via Major Histocompatibility Complex (MHC) molecules. Presentation on MHC class I molecules activates CD8^+^ cytotoxic T cells, leading to the elimination of infected cells. Conversely, presentation on MHC class II molecules activates CD4^+^ helper T cells, stimulating antibody production by B cells and initiating a broad immune response [145]. For example, mice administered with IAV NP-encoding mRNA in liposome complexes demonstrated strong cytotoxic T-cell responses [146].

Additionally, the mRNA triggers an innate immune response through its recognition by dendritic cell pattern recognition receptors such as Toll-like receptors (TLR3, TLR7/8) and RIG-I/MDA5. This stimulates the production of type I interferons and other cytokines, enhancing the immune response through both autocrine and paracrine actions, which increase the expression of MHC and costimulatory molecules. This comprehensive immune activation not only targets the immediate threats but also fosters the development of memory T and B cells, crucial for long-term immunity [146,147]. In summary, mRNA vaccines harness cutting-edge technology to simulate natural infections and stimulate an extensive immune response, providing a versatile and dynamic approach to preventing influenza and preparing for future pandemics.

#### 3.2.4. Safety and Stability

The production system for mRNA vaccines is a standardized, cell-free process [130,148]. This approach not only reduces the risk of bacterial contamination but also avoids the ethical and logistical issues associated with animal-derived products. mRNA vaccines are inherently safe; they contain no live virus and do not integrate into the host genome, eliminating risks of infection or genetic mutation.

They degrade naturally in the cell, and their stability can be controlled through various modifications and delivery methods, such as improved LNP formulations. These advancements not only increase the thermal stability of mRNA vaccines but also simplify their distribution and storage, making them more accessible for global vaccination campaigns [138]. Additionally, the production of mRNA-LNP eliminates the need for egg-based production processes. This aspect of mRNA vaccine production ensures that individuals with egg allergies are not at increased risk when receiving these vaccines [149].

### 3.3. Applications of mRNA Vaccines in Pulmonary Influenza

#### 3.3.1. Animal Studies

Animal studies have shown that mRNA vaccines targeting specific influenza HA antigens have yielded high antibody titers and significantly lower viral loads in the lungs and boost survival rates following influenza exposure. For instance, ferrets immunized with mRNA vaccines targeting conserved influenza antigens demonstrated enhanced cellular responses in the lungs and nasal passages [150,151]. Notably, a quadrivalent mRNA vaccine designed for four major seasonal influenza strains has shown high immunogenicity and effectiveness [123]. Moreover, mRNA vaccines have elicited cross-reactive immune responses, proven by a pentavalent vaccine’s success in shielding against several strains of influenza B virus [152]. These vaccines are tailored to address both specific subtypes and broader influenza categories, targeting prevalent subtypes like H1N1 and H3N2 with strain-specific antigens (e.g., A/California/07/2009 and A/Hong Kong/4801/2014) [134,153], as well as both Victoria and Yamagata lineages of influenza B with strains like B/Brisbane/60/2008 and B/Phuket/3073/2013 [154,155].

To enhance immunogenicity further, some vaccines include conserved proteins such as nucleoprotein (NP) and matrix protein 1 (M1) [156], enhancing the immune response and offering potential cross-protection across different influenza subtypes [157]. This approach aims to develop a more universal vaccine capable of defending against a wide spectrum of influenza strains, thereby addressing variations that might bypass more narrowly focused vaccines.

#### 3.3.2. Clinical Trials

Animal studies have demonstrated that mRNA vaccines can induce robust immune responses, significantly reducing viral titers in the lungs and improving survival rates after viral challenges. These promising results in preclinical models have paved the way for clinical trials. Moderna’s quadrivalent mRNA vaccine (mRNA-1010) is a notable example currently under clinical evaluation [120]. It aims to provide broad-spectrum protection against multiple influenza strains, demonstrating the adaptability of mRNA vaccine technology, showing dose-dependent protection against lethal challenges from multiple influenza subtypes, highlight the potential of these vaccines to serve as universal protectants against influenza [120,121].

Expanding the range of protection, Moderna has also developed several seasonal influenza vaccine candidates—mRNA-1011.1, mRNA-1011.2, and mRNA-1012.1—currently undergoing phase I/II trials to address different circulating strains [122]. Additionally, mRNA-1083, targeting both influenza and COVID-19, exemplifies the potential of mRNA technology to concurrently address multiple respiratory pathogens. However, it is important to note that to date, mRNA vaccines have been prominently tested during the COVID-19 pandemic, with varied efficacy rates. For instance, the efficacy rate for the 2023–2024 season in the US was approximately 58%, highlighting the vaccine’s role in public health but also emphasizing the need for ongoing evaluation and development to optimize their performance across different settings and virus strains [122].

Despite the initial high hopes, Pfizer and BioNTech’s dual COVID-19 and influenza vaccine did not meet a primary endpoint in a Phase III trial, particularly failing to show non-inferiority against the influenza B strain, although it was effective against influenza A (NCT06178991). This mixed result has led to a strategic reassessment of the vaccine’s development path. Similarly, GSK’s candidate, GSK4382276A, is in phase I/II trials to evaluate its effectiveness against specific influenza subtypes [125].

## 4. Limitation and Future Perspectives

mRNA vaccines have demonstrated exceptional efficacy and swift deployment during recent global health emergencies, highlighting their potential for managing influenza outbreaks. These vaccines can be rapidly customized to match current viral strains, addressing a major challenge in influenza vaccine design. Furthermore, mRNA vaccines offer significant safety advantages over traditional vaccines, reducing risks and making them suitable for widespread use, including in populations with compromised health [142]. Such a vaccine would target conserved regions of the virus, potentially offering durable protection against multiple strains. Furthermore, the versatility of mRNA technology could lead to the creation of combination vaccines that address several respiratory pathogens simultaneously, such as influenza, RSV, and SARS-CoV-2. These vaccines could significantly lessen the overall impact of respiratory diseases [158,159].

Although mRNA vaccines, particularly those utilizing lipid nanoparticles (LNPs), have demonstrated robust immune responses, they also present specific safety concerns that merit attention. Studies comparing mRNA vaccines to recombinant protein vaccines have shown that both elicit high levels of specific binding and neutralizing antibodies [160]. However, mRNA vaccines tend to provoke more intense cellular immune responses, which, in very rare cases, can lead to adverse events such as myocarditis and pericarditis—particularly after the second dose in younger males [161]. This has led health authorities to issue warnings; however, these adverse events remain significantly rarer than similar or more severe complications associated with severe COVID-19 infection. Notably, the risk of cardiac outcomes after SARS-CoV-2 infection is 1.8 to 5.6 times higher than after the second vaccine dose [162,163]. The ionizable cationic lipids in LNPs are known to activate various inflammatory pathways, potentially heightening the reactogenicity seen with these vaccines compared to recombinant protein vaccines, which typically cause more localized reactions at the injection site [164]. These adverse events are thought to originate from the innate immune activation triggered by the vaccine’s LNP formulation, resulting in systemic inflammatory responses, including pain, swelling, and fever [165]. Such reactogenicity may escalate in very rare instances to severe clinical conditions like myocardial infarction, Bell’s palsy, cerebral venous sinus thrombosis, and Guillain–Barré syndrome [166]. However, it is crucial to note that these serious adverse events are far less frequent than the severe complications associated with COVID-19 infection, which can lead to long-term organ damage, respiratory failure, and even death [167,168].

Additionally, concerns have been raised regarding the role of IgG4 in immune response modulation after repeated mRNA vaccinations. Investigations have detected elevated IgG4 levels in individuals receiving multiple doses of mRNA vaccines, a pattern also observed with vaccines against HIV, malaria, and pertussis [169]. However, such elevations in IgG4 are exceptionally rare and have not been conclusively linked to widespread adverse outcomes.

Technological innovations in nanoparticle formulations and delivery methods are significantly enhancing the stability and efficacy of mRNA vaccines. These advancements reduce the need for stringent cold chain logistics, enabling broader distribution, particularly vital in resource-limited settings. Active development in the pharmaceutical formulation of mRNAs is ongoing, with efforts focused on creating stable formulations at higher temperatures to facilitate easier distribution. For example, the RNActive platform has demonstrated stability after lyophilization, remaining active at temperatures ranging from 5 to 25 °C for up to three years and at 40 °C for six months. Similarly, freeze-dried naked mRNA has been shown to remain stable under refrigerated conditions for at least 10 months. Enhancements such as nanoparticle packaging or co-formulation with RNase inhibitors are being explored to further improve the stability of mRNA vaccines. Further exploration is needed into how their immunogenicity—their capacity to provoke an immune response—can be modulated to maximize efficacy and safety [170]. By controlling the cellular half-life of the mRNA and its presentation within the body, scientists can fine-tune the vaccine’s performance and safety profile. This allows for the tailored adjustment of the vaccine’s properties to meet specific health needs [171,172].

As we advance the deployment of mRNA vaccines, addressing public perceptions that affect vaccine uptake becomes paramount. These vaccines, including those developed for diseases like influenza, have proven effective; however, they encounter considerable hesitancy. Such reluctance primarily stems from misconceptions about the technology and anxieties concerning long-term safety. Addressing these issues necessitates robust patient education programs. These initiatives must clearly and transparently articulate the benefits and safety of mRNA vaccines to counteract misinformation and foster community trust. For achieving extensive vaccination coverage and effective disease control, it is imperative to launch comprehensive public education campaigns. These should aim to clarify how mRNA vaccines work, detail their safety profiles, and outline their health benefits, thus dispelling prevalent myths. Enhancing public understanding and acceptance of these vaccines involves collaborative efforts with community leaders, healthcare providers, and media outlets to spread accurate and helpful information.

In conclusion, mRNA vaccines not only provide a promising solution to current influenza challenges but also represent a versatile platform that could revolutionize vaccine development and deployment for various infectious diseases.

## Figures and Tables

**Table 2:** Current status of mRNA vaccines for the Influenza virus in clinical trials.

Vaccine	Antigen	Delivery Method	Trial Status	Effects	Sponsor/Collaborators	References
mRNA-1440	pre-membrane and E protein of H10N8 influenza virus	LNP	Completed Phase 1 clinical trials	Inducing strong humoral immune response against the H10N8 influenza virus	Moderna	[117,118]
mRNA-1851	HA of H10N8 influenza virus	LNP	Completed Phase 1 clinical trials	Inducing strong humoral immune response against the H10N8 influenza virus	Moderna	[119]
mRNA-1010	HA of H1N1, H3N2, B/Victoria, B/Yamagata *	LNP	Currently in Phase 3 clinical trials	Achieving higher antibody responses for A/H3N2 and A/H1N1; less effective for B strains. More efficacy data expected	Moderna	[120,121]
mRNA-1083	HA of H1N1, H3N2, B/Victoria, and SARS-CoV-2	LNP	Currently in Phase 3 clinical trials	Combines mRNA-1010 with mRNA-1283 achieving higher antibody responses for three targeted strains and SARS-CoV-2 virus	Moderna	[122]
Influenza modRNA	HA of H1N1, H3N2, B/Victoria, B/Yamagata *	LNP	Completed 3 Phase 2 trials	Inducing protective antibody titers against four targeted strains	Pfizer	[123,124]
GSK4382276A	HA of H1N1, H3N2, B/Victoria, B/Yamagata *	LNP	Currently in a second Phase 2 trial	Specific clinical results have not yet been released	GSK	[125]
Sanofi Quadrivalent mRNA Vaccine	HA of H1N1, H3N2, B/Victoria, B/Yamagata *	LNP	Completed 7 Phase 1 clinical trials	Inducing protective antibody titers against four targeted strains	Sanofi	[15,123]
MRT5400	HA of H1N1, H3N2, B/Victoria, B/Yamagata *	LNP	Currently in Phase 1 and Phase 2 clinical trials	Have not been publicly released	Sanofi	-
MRT5401	HA of H3N2	LNP	Currently in Phase 1 clinical trials	Have not been publicly released	Sanofi	-

* Note: Since the B/Yamagata lineage has been declared extinct, it is recommended to reevaluate its inclusion in forthcoming vaccine updates to ensure that formulations reflect the latest epidemiological findings and maintain their efficacy.
